# Nervous Functional Diseases Due to the War

**Published:** 1918-03

**Authors:** 


***Nervous Functional Diseases Due to the War.*** By Dr. SCHNYDER of Berne. Abs. by J. M. WOLFSOHN from *Schweizer Archiv für Neurologie und Psychiatrie,* Bd. II, Heft I, Zurich, 1918.

The article is largely the result of observations made during a tour of the neurological and psychiatrical military centers of Lyons and Paris. The author gives a comprehensive report of the opinions of most authorized French representatives of psycho-neuropathology in those centers.

He is much impressed by the work of Sollier, who attributes most of the disorders in question to Charcot's classical Hysteria and sees in them the confirmation of his theories of the physiological origin of hysteria. Sollier further lays great stress on the acuteness of the deep joint sensibility, and on the fact that massive anesthesia of the limbs, attacked by flaccid paralysis or contracture does not account for psychic causes and suggestion, as Babinski affirms, but is responsible for a cerebral disturbance and for a state of inhibition of certain cerebral centers. Sollier, in his treatment, tries to arouse the inhibited centers by inducing in the patient painful sensations by means of an energetic motor re-education of the affected extremities, consisting of passive movements of the affected limbs. Many soldiers, however, seem to object to this treatment.

The author agrees that many of these patients are neuropathic, and a psychic element is important in the genesis of the functional disorders. He quotes Sollier as saying : " True simulation is very rare, but cases of exaggeration of symptoms, such as pain, stiff joints, and vicious attitudes, headache, vertigo, and functional impotencies, are more frequent and that, just as it is, practically speaking, impossible to simulate a syndrome, so is it easy to keep it up, once it exists ". The causes of this perseverance are : *Apprehension, indifference, unwillingness, ignorance of what to do for a cure, certain untimely treatments*, reducing apparatus, bandages applied inopportunely or kept on too long because of the zeal of far too sympathetic nurses.

Sollier further remarks that in 561 cases, 259 occurred in the first six months of the war, 271 in 1915 and in nine months of 1916. Neither the Battle of Verdun nor the French offensives have increased the number of these cases. He concludes from these facts that at the beginning of the War, the unfavorable moral condition of the French soldier, his physical exhaustion and lack of sufficient medical care, were the chief causative factors of the neuroses. Sollier accounts for all the phenomena in true " shock " cases on physical grounds. The physical effects are due either to the instantaneous combustion or to the phenomena of vibration. He says that the disturbances resulting from the shock are either phenomena of inhibition or of exhaustion due to excessive excitement of the sensory organs and the central nervous system or to a molecular disturbance caused by vibrations in the colloidal equilibrium of the nerve cells.

George Dumas sees, in all " shock " cases, a state of mental confusion more or less pronounced which favors auto-suggestion and the production of certain nervous disturbances.

Ernest Dupree says that in the production of complications following shock, which are the same in civil life as in war, the succession of factors are: commotion, emotion, suggestion, exaggeration, simulation, and claim (pension).

Even at this time it is permissible to say that the war has not created any new type of psychosis or psycho-neurosis. It has simply multiplied and amplified these cases.

Among the French neurologists, the psychic factor in the pathogeny of these disorders has gained in importance.

In considering the therapeutics of war psychoneuroses, psychotherapy in its different forms claims an important rôle. It is often associated with physical methods like electricity, to strengthen the verbal suggestive methods or to work a kind of contra-commotion with certain functions as in cases of post-shock deaf-mutism.

According to the author, Sicard does not account for the functional contractures by hysteria or pithiatism. He calls them "acromyotonia" (muscular hyperkinesiae) and he sees in them analgesic attitudes directed against pain.

In pointing out the importance of the psychic factor in the production of traumatic hysterical complications, Dr. Maurice Dide points out "that in a battalion of Alpine Chasseurs, functional motor disturbances in fighting units which have energetic officers in command, are an extreme rarity". He says further that "The war has convinced me that neither incessant privations, nor overwhelming emotions can cause these phenomena in the vast majority of men who possess the strong belief that they are as brave as anybody."

As to the causes of neuroses and mental disorders following detonation of high explosives: Dr. A. Vincent in a report to the Paris Neurological Society makes a distinction between:

(1) emotional phenomena, (2) commotional phenomena, (3) mental phenomena.

In (1) the man has not lost consciousness and is capable of flight.

In (2) the man has lost consciousness for a variable time and has to be carried.

In (3) the man is not unconscious but is often mentally absolutely inert or else possesses emotive phenomena, e. g., delirium, hallucinations, etc.

Roussy and others say that this classification is too arbitrary and that the three are no longer different.

Henry Claude points out two kinds of shock.

(1) By traumatism to the skull or spinal column causing a nervous lesion (direct commotion).

(2) Indirect commotion from the explosion without traumatism, with symptoms consisting of general phenomena without signs of nervous lesions, e. g., nervous depression, general torpor, psychic slowness, amnesia, emotivity, sleep disorders, hyperacousia, psycho-neuropathic disorders and hysterical manifestations.

He has never seen in recent cases the presence of blood in the arachnoidal cavity, but hypertension — 23 to 50 cm. water — was found in 18 cases and hyperalbuminosis in 10 cases. He also believes that commotion, caused by explosives, very seldom produces organic lesions in the nervous centres.

The author impresses his readers with the statement made by Dr. André Thomas: " Psychotherapy, i. e., rational psychotherapy, is a system for peace times ". He says that the great drawback now is the lack of time at the disposal of the doctors.

In conclusion, the author describes a method of " psychic isolation " used by Prof. Landau which has apparently worked well with some very resistent hysterical cases, but he finishes by saying that the maximum effect of rational psychotherapy will be gained in the sanitary formations at the front at the onset of the psycho-nervous troubles.

